# Lysophosphatidic acid induces integrin activation in vascular smooth muscle and alters arteriolar myogenic vasoconstriction

**DOI:** 10.3389/fphys.2014.00413

**Published:** 2014-10-31

**Authors:** Marius C. Staiculescu, Francisco I. Ramirez-Perez, Jorge A. Castorena-Gonzalez, Zhongkui Hong, Zhe Sun, Gerald A. Meininger, Luis A. Martinez-Lemus

**Affiliations:** ^1^Dalton Cardiovascular Research Center, University of MissouriColumbia, MO, USA; ^2^Department of Bioengineering, University of MissouriColumbia, MO, USA; ^3^Department of Medical Pharmacology and Physiology, University of MissouriColumbia, MO, USA

**Keywords:** focal adhesion, basal tone, myogenic response, integrin activation, fibronectin, reactive oxygen species

## Abstract

In vascular smooth muscle cells (VSMC) increased integrin adhesion to extracellular matrix (ECM) proteins, as well as the production of reactive oxygen species (ROS) are strongly stimulated by lysophosphatidic acid (LPA). We hypothesized that LPA-induced generation of ROS increases integrin adhesion to the ECM. Using atomic force microscopy (AFM) we determined the effects of LPA on integrin adhesion to fibronectin (FN) in VSMC isolated from rat (Sprague–Dawley) skeletal muscle arterioles. In VSMC, exposure to LPA (2 μM) doubled integrin-FN adhesion compared to control cells (*P* < 0.05). LPA-induced integrin-FN adhesion was reduced by pre-incubation with antibodies against β_1_ and β_3_ integrins (50 μg/ml) by 66% (*P* < 0.05). Inhibition of LPA signaling via blockade of the LPA G-protein coupled receptors LPAR1 and LPAR3 with 10 μM Ki16425 reduced the LPA-enhanced adhesion of VSCM to FN by 40% (*P* < 0.05). Suppression of ROS with tempol (250 μM) or apocynin (300 μM) also reduced the LPA-induced FN adhesion by 47% (*P* < 0.05) and 59% (*P* < 0.05), respectively. Using confocal microscopy, we observed that blockade of LPA signaling, with Ki16425, reduced ROS by 45% (*P* < 0.05), to levels similar to control VSMC unexposed to LPA. In intact isolated arterioles, LPA (2 μM) exposure augmented the myogenic constriction response to step increases in intraluminal pressure (between 40 and 100 mm Hg) by 71% (*P* < 0.05). The blockade of LPA signaling, with Ki16425, decreased the LPA-enhanced myogenic constriction by 58% (*P* < 0.05). Similarly, blockade of LPA-induced ROS release with tempol or gp91 ds-tat decreased the LPA-enhanced myogenic constriction by 56% (*P* < 0.05) and 55% (*P* < 0.05), respectively. These results indicate that, in VSMC, LPA-induced integrin activation involves the G-protein coupled receptors LPAR1 and LPAR3, and the production of ROS, and that LPA may play an important role in the control of myogenic behavior in resistance vessels through ROS modulation of integrin activity.

## Introduction

Integrin activation and signaling are indispensible for cell survival, proliferation and motility under physiological and pathological conditions. Recently, increased integrin adhesion has been associated with aging and hypertension (Zhu et al., 2012; Sehgel et al., 2013). Integrins are heterodimeric cell surface receptors composed of one α and one β subunit that are non-covalently associated. Their N-terminal domains mediate attachment to the extracellular matrix (ECM), while their intracellular domains attach to a series of scaffolding proteins that ultimate connect to the cytoskeleton (Martinez-Lemus et al., 2003). Integrins do not possess enzymatic activity, but many of the scaffolding proteins that attach to their intracellular domains do. This allows integrins to transmit signals from the ECM to the cytoskeleton (outside-in) and vice versa (inside-out), making them particularly suited to participate in the mechanotransduction of signals across the plasma membrane of many cell types (Wilson et al., 1995; Goldschmidt et al., 2001; Balasubramanian et al., 2007; Delon and Brown, 2007; Hong et al., 2012). In vascular smooth muscle cells (VSMC), integrin activity has been associated with a number of physiological and pathological processes including the modulation of myogenic phenomena and vascular remodeling (Martinez-Lemus et al., 2005a; Heerkens et al., 2006). In particular, integrins have been postulated to serve as mechanosensors able to convert changes in physical parameters such as ECM tension and cellular stress into chemical signals that modulate vascular diameter (Martinez-Lemus et al., 2005b). Previously, we have shown that α_v_β_3_ and α_5_β_1_ integrin blockade inhibits the myogenic vasoconstriction associated with increments in intravascular pressure in isolated arterioles (Martinez-Lemus et al., 2005a). We also showed that in rat VSMC, lysophosphatidic acid (LPA) increases integrin fibronectin (FN) adhesion, suggesting that LPA modulates integrin activation (Sun et al., 2005). Additionally, transforming growth factor-β activation, associated with airway remodeling in severe asthma, is induced by exposure to LPA and is preceded by integrin activation (Tatler et al., 2011). These studies in isolated arterioles and smooth muscle cells (SMC) clearly indicate that mechanical forces and LPA modulate integrin activity. However, the exact mechanisms by which mechanical forces and in particular LPA induce integrin activation are not completely known.

LPA is a small phospholipid with a wide array of biological functions such as, cell proliferation and survival, platelet aggregation, cell migration, and SMC contraction (Cheng et al., 2009; Cui, 2011; Gaaya et al., 2012; Nam et al., 2013). The biological effects of LPA are mediated through specific G-protein coupled cell surface receptors named LPAR1-6 (Choi et al., 2010; Yanagida and Ishii, 2011) and through intracellular receptors such as the peroxisome proliferator-activated receptor-γ (Cheng et al., 2009). *In vivo*, administration of LPA results in acute increases in blood pressure in different animal species (Tokumura et al., 1985, 1995), suggesting a role for LPA in both normal blood pressure regulation and hypertension. Furthermore, LPA mediated signaling has been shown to be involved in neointimal proliferation (Cheng et al., 2009), vascular remodeling (Yoshida et al., 2003), and angiogenesis (Sumida et al., 2010). Recent studies also indicate that, in VSMC, LPA is responsible for the generation of reactive oxygen species (ROS). In one study, the mitogenic effects of LPA were diminished by the inhibition of nicotinamide adenine dinucleotide phosphate (NADPH) oxidase (Schmitz et al., 2002). Similarly, LPA induced proliferation of human aortic VSMC is dependent on the activity of Rac-1 and is blocked by treatment with antioxidants or inhibition of NADPH oxidase (Kaneyuki et al., 2007). These studies indicate that LPA-stimulation of cells results in increased ROS production. Therefore, we designed this study to test the hypothesis that, in VSMC, LPA activates integrins and increases integrin-FN adhesion through pathways that depend on the production of ROS. As mentioned above, we have previously shown that integrins are involved in the myogenic response (Martinez-Lemus et al., 2005a). Recently, myogenic vasoconstriction as it occurs in response to increments in intraluminal pressure in resistance arteries has been associated with the production of ROS by cells within the vascular wall (Gebremedhin et al., 2013). Therefore, we also tested the effects of LPA on myogenic reactivity in isolated resistance vessels expecting that myogenic vasoconstriction would be enhanced via LPA-dependent production of ROS.

## Materials and methods

### Animal model

All procedures involving animals complied with the NIH Guide for the Care and Use of Laboratory Animals, and were approved by the Animal Care and Use committee of the University of Missouri-Columbia. Male Sprague-Dawley rats (180–250 g) were anesthetized by an intraperitoneal injection with 100 mg/Kg pentobarbital sodium. Following anesthesia, the cremaster muscle was excised through a scrotal incision. After excision of the tissue, the rat was euthanized by bilateral pneumothorax and exsanguination.

### VSMC isolation and culture

VSMC were enzymatically isolated from first-order cremasteric arterioles using previously described methods (Sun et al., 2008). Briefly, VSMC were isolated from first order cremasteric arterioles via enzymatic digestions with papain, collagenase, and elastase. The isolated VSMC were maintained in culture conditions in DMEM/F-12 media (Invitrogen, Carlsbad, CA) supplemented with 10% fetal bovine serum (FBS, Atlanta Biologicals, Lawrenceville, GA), 100 μg/mL streptomycin, 100 U/mL penicillin, 2 mM L-glutamine, 1 mM sodium pyruvate, and 10 mM 4-(2-hydroxyethyl)-1-piperazineethanesulfonic acid (HEPES, Sigma, St Louis, MO, USA). Cells were plated on 60 mm tissue culture dishes (Falcon, BD Labware, Lincoln, NJ) and kept in a humidified incubator (Heraeus Instruments, Newtown, CT) with 5% CO_2_ at 37°C. In all experiments cells at passages 3–13 were used. Except were otherwise indicated, the above reagents and chemicals were purchased from Invitrogen.

### Integrin adhesion assays

All adhesion assays were performed using an MFP-3D atomic force microscopy (AFM) system (Asylum Research, Santa Barbara, CA) mounted on an inverted Olympus IX81 optical microscope. Data was collected using Igor Pro 6.0 (WaveMetrics, Lake Oswego, OR) and analyzed using a software package written in Matlab (MathWorks, Natick, MA) (Sun et al., 2005). All AFM probes were functionalized with FN. For this purpose, a spherical 5 μm borosilicate micro bead (Spi Supplies, West Chester, PA, USA) was attached to a triangular silicon-nitride AFM probe (Veeco-MLCT 010, spring constant = 30 pN/nm) using epoxy glue. Subsequently, the AFM probe was functionalized with FN (Invitrogen, Carlsbad, CA) as we have previously described (Qiu et al., 2010; Hong et al., 2012). Briefly, 10 mM polyethylene glycol (Sigma, St. Louis, MO) was used as a non-covalent linker to facilitate the passive absorption of FN onto the borosilicate glass micro bead. The probe was first incubated 5 min with polyethylene glycol, washed 3 times with phosphate buffered saline, and then incubated 5 min with FN (0.2 mg/ml). Following the FN incubation, the AFM probe was washed 3 times with phosphate buffered saline. Calibration experiments revealed that the functionalization procedure did not significantly alter the spring constant of the AFM probe.

**Figure 1:**
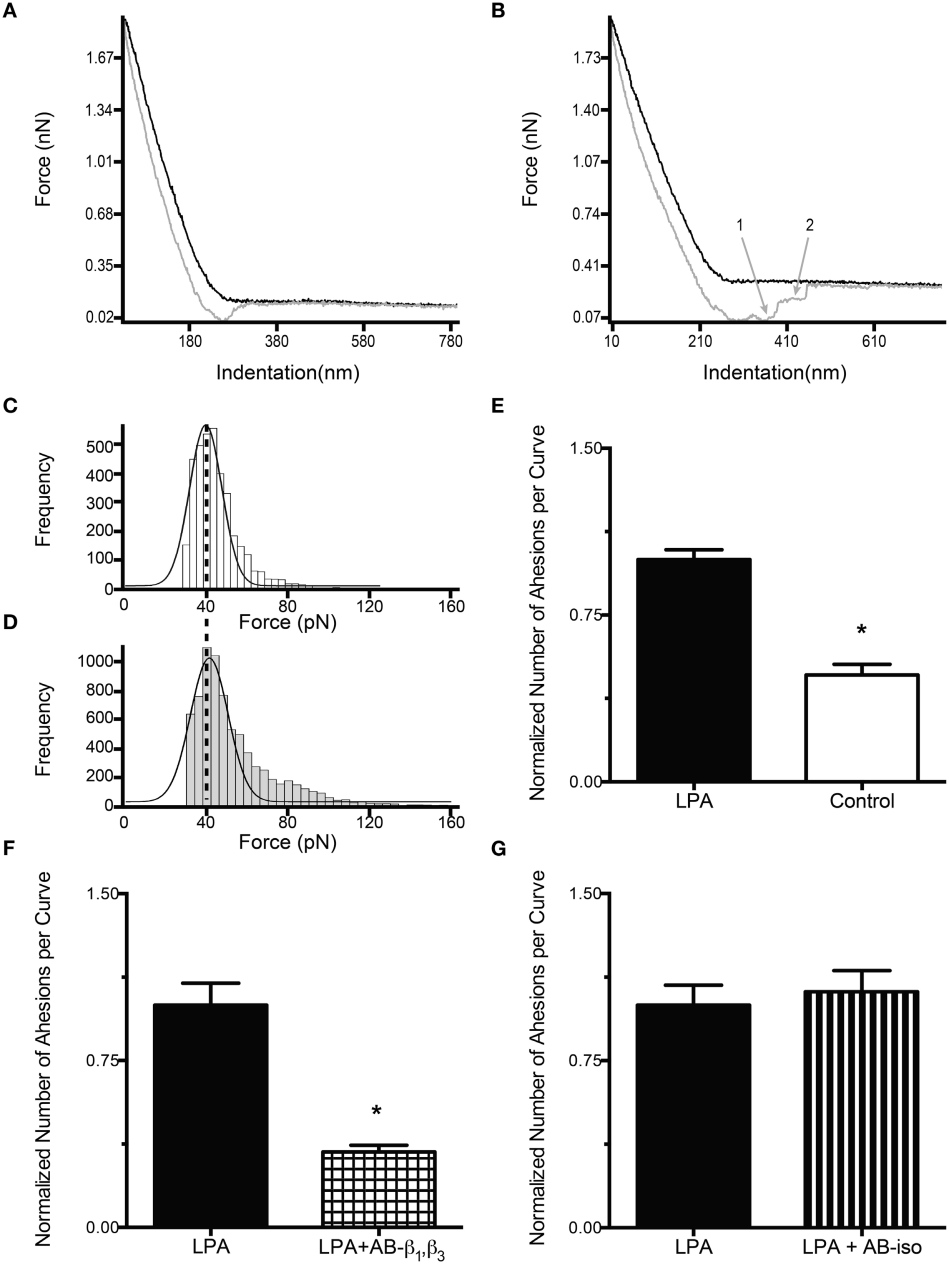
**LPA increases the adhesion between FN and integrins containing β_1_ and β_3_ subunits. (A–B)** Typical force curves obtained during AFM adhesion experiments. The black line represents the cantilever approach curve, while the gray one represents the retraction curve. The numbers on gray curves represent adhesion events detected as sharp vertical jumps in the retraction curve. **(A)** Force curve with no adhesion events. **(B)** Force curve with 2 adhesion events (1, 2). Force adhesion events distribution for control **(C)** and LPA treated cells **(D)**. The number of single adhesion events (first peak) in the LPA treated cells **(D)** increases significantly above the control level **(C)**. The adhesion force is not changed by LPA treatment. **(E)** VSMC adhesion to FN is reduced significantly, by 52%, in untreated (control) cells (*n* = 40) compared to LPA treated cells (*n* = 40). **(F)** The incubation of VSMC with LPA (2 μM) in the presence of β_1_ and β_3_ (50 μg/ml) antibodies (*n* = 40) resulted in a 66% decrease in the number of adhesion events per curve compared to VSMC treated with LPA alone (*n* = 40). **(G)** Compared with the VSMC treated with LPA alone (*n* = 40), LPA treatment (2 μM) in the presence of isogroup control antibody (50 μg/ml), does not significantly change the number of adhesion events per curve (*n* = 38). Data are means ± s.e.m. ^*^P < 0.05 vs. LPA **(E,F)**.

Real-time monitoring of the FN coated bead adhesive properties on VSMC was performed with the AFM in force mode by repeated nanoindentation (black curve, Figures 1A,B) and retraction (gray curve, Figures 1A,B) from the cell surface at a 0.5 Hz sampling frequency, ~800 nm/s scanning speed, and 800 nm force distance. For each experiment, cells were randomly selected and probed at sites located 1/3–1/2 the distance between the cell margin and the nucleus. One hundred force curves for each cell were collected over a period of ~3.5 min up to a maximum of 10 cells per dish. During retraction of the AFM probe, the lack of adhesion events results in the retraction curve closely following the approach curve without abrupt changes in the force required to pull the cantilever away from the cell (Figure 1A). Alternatively, the presence of adhesion events is detected as sharp vertical shifts in the retraction curve (Sun et al., 2005) (Figure 1B). The number of adhesions per curve was quantified as the total number of unbinding (adhesion) events detected for one cell, divided by the total number of force-curves recorded for the same cell. All the AFM experiments were performed at room temperature. To minimize the effects of drift, the system was equilibrated mechanically and thermally for at least 30 min with the probe submerged in serum-free media. For each experiment the same probe was used on control and treated cells. The sampling order was randomized between controls and treatments.

To determine the effect of LPA on the adhesion of FN to VSMC integrins, cells were exposed to LPA (2 μM) in serum free DMEM/F-12 media for 2 h at 37°C in 5% CO_2_. Subsequently, cells were transferred to the AFM and the adhesion properties were determined as described above. Control cells were exposed to serum free media in the absence of LPA. Exposure to LPA was maintained for the duration of the AFM experiments.

To determine if the effect of LPA on adhesion events occurred via LPA signaling through VSMC membrane bound G-protein coupled receptors, LPA receptors (LPAR) were blocked with Ki16425 (10 μM). Ki16425 (Selleckchem, Houston, TX) was added to the cells 30 min before and throughout the exposure of cells to LPA. Control cells for this series of experiments did not receive Ki16425, but were exposed to LPA (2 μM) as described above.

To corroborate that the changes in VSMC adhesion to the FN coated bead was mediated by integrins, specific function-blocking monoclonal antibodies against β_1_ (50 μg/mL, Ha 2/5) and β_3_ (50 μg/mL, F11) integrins were used. The antibodies were added simultaneously to the cell bath 30 min before the AFM experiments were performed while the VSMC were incubated in serum free media in the presence of LPA (2 μM). Control cells were exposed to an isotype control antibody (50 μg/mL, G235-1). Additional control experiments were performed in parallel without antibodies in the presence of LPA (2 μM). All antibodies were purchased from BD Pharmingen (San Jose, CA).

To determine the role of ROS on the changes in integrin to FN adhesion induced by LPA, AFM experiments were performed in the presence of the superoxide dismutase mimetic, tempol or the NADPH oxidase inhibitor, apocynin (Calbiochem, Billerica, MA). Tempol (250 μM), apocynin (300 μM) or DMSO (5 mg/ml), were added to the cells 30 min before and throughout the 2-hour exposure to LPA. Additional control experiments were performed in parallel without LPA.

### ROS detection assay

To corroborate that LPA increases the production of ROS in VSMC, we used the ROS-sensitive fluorescent probe dihydroethidium (DHE) to assess the presence of intracellular ROS. In these experiments, VSMC plated in glass bottom dishes (Wilco Wells, Amsterdam, Netherlands) were incubated in serum free media for 2 h in the presence or absence (control) of LPA (2 μM). DHE (10 μM) was applied to the serum free media concurrently with LPA or its vehicle control. After the application of DHE, the cells were imaged with a confocal microscope (Leica TCS SP5) using a 40X oil-immersion objective (numerical aperture 1.25). During imaging, the cells were kept at room temperature as in the AFM experiments. Images obtained after 2 h of incubation with LPA or vehicle control were compared for fluorescence intensity. All images were obtained with exactly the same parameter settings: excitation wavelength 514 nm, emission detection wavelength range 600–700 nm, and detector gain of 600. A total of 42 z-sections were acquired at 0.5 μm intervals covering the complete height of the cells. The mid cell section was used for image analysis. A Matlab script was used to analyze the images. A background identification and characterization was performed in each picture via analysis of pixel intensity distribution. Analysis of the lowest intensity pixels allowed us to measure the mean lowest intensity and its corresponding standard deviation in areas outside the cell. These parameters were used to set a dynamic threshold and perform background subtraction. Pixels above threshold were identified as being part of cell material through automated selection criteria. Total cell area (total number of pixels), total cell intensity (sum of the intensity for all pixels above threshold) and total cell volume (total area times total intensity) were computed. In order to normalize the signal by the amount of cell material in each picture (cell size and number of cells), ratios between total cell intensity and total cell area were calculated and compared for control and treated samples.

In an additional series of experiments, VSMC were incubated with Ki16425 (10 μM) to determine the effect of LPAR inhibition on the production of ROS. The Ki16425 inhibitor was added to the cells 30 min before and throughout the exposure to LPA and DHE. Additional control experiments for this series were performed in the presence of LPA and DHE without Ki16425.

### Isolated vessel experiments

First-order cremaster arterioles were isolated, cannulated, and pressurized to monitor vascular diameter changes *ex vivo* as previously described (Martinez-Lemus et al., 2011). To this end, the excised cremaster muscle was pinned flat in a refrigerated (4°C) dissecting chamber and a first-order segment of the cremaster feed arteriole was isolated. The dissection chamber contained physiological saline solution (PSS) of the following composition: 5.0 mM dextrose, 3.0 mM 3-(*N*-morpholino) propane sulfonic acid (MOPS) buffer, 2.0 mM pyruvate, 0.02 mM EDTA, 145.0 mM NaCl, 4.7 mM KCL, 2.0 mM CaCl_2_, 1.2 mM MgSO_4_, 1.0 mM NaH_2_P0_4_, and 0.15 mM bovine serum albumin, at pH 7.4. The isolated arterioles were cannulated, without flow at 60 mm Hg in an observation chamber (Living Systems Instrumentation, Burlington, VE) filled with MOPS-PSS without albumin. The chamber was mounted on an inverted microscope (Zeiss Axiovert 40 C). The luminal diameter was monitored with a video caliper system connected to a video camera and the changes in the inner diameter were recorded using Lab Chart 7 (ADInstruments, Colorado Springs, CO) as previously described (Martinez-Lemus, 2008).

To determine the effect of LPA on myogenic constriction, resistance arteries were exposed for 2 h to LPA (2 μM). Control vessels were exposed to PSS in the absence of LPA. Then arterioles with or without LPA were exposed to 20 mm Hg incremental changes in intravascular pressure from 40 to 100 mm Hg. Exposure to LPA was maintained during the acquisition of the pressure diameter curves.

To determine if the effect of LPA on myogenic constriction occurred via LPA signaling through VSMC membrane bound G-protein coupled receptors, LPA receptors (LPAR) were blocked with 10 μM Ki16425. At this concentration, Ki16425 preferentially inhibits LPAR1 and LPAR3-mediated responses and has moderate inhibitory effects on LPAR2 (Ohta et al., 2003). Ki16425 was added to the vessels 30 min before and throughout the exposure of vessels to LPA. In this series of experiments, Ki16425 was dissolved in methanol. The final concentration of methanol in the PSS buffer was 0.25% v/v.

To determine how ROS modulate the effects of LPA on myogenic constriction, pressure-diameter experiments with or without LPA were performed in the presence of the superoxide dismutase mimetic, tempol (Calbiochem, Billerica, MA), or the cell permeable NADPH oxidase inhibitor, gp91 ds-tat (Biomatik, Wilmington, DE). In vessels, we decided to use gp91 ds-tat instead of apocynin to inhibit NADPH oxidase production of superoxide because in our experience dimethyl sulfoxide (DMSO), the chemical used to dissolve apocynin, causes vessels to develop vasomotion through an endothelium-dependent mechanism. This makes it difficult to determine with precision when a stable diameter is reached after a change in intraluminal pressure. Tempol (250 μM) was added to the vessels 30 min before and throughout the 2-hour exposure to LPA, while gp91 ds-tat (50 μM) was added 30 min before and throughout the first 30 min of incubation with LPA. Total incubation time with LPA was 2 h.

### Statistical analysis

Data are reported as means ± s.e.m. Statistical analysis for all the AFM and confocal microscopy data was performed using two-tailed, unpaired Student's *t*-test. The slopes of the myogenic constriction were analyzed as previously described (Martinez-Lemus et al., 2005a). Briefly, using linear regression analysis we calculated the slopes of the changes in diameter induced by the step increases in pressure. The average slope values were analyzed using two-tailed unpaired Student's *t*-test, for comparisons between two groups, and One-Way ANOVA followed by Bonferroni correction for multiple comparisons, for comparisons between multiple groups. Values refer to the number of cells from each group or the number of vessels used to calculate mean values. A value of *P* < 0.05 was considered statistically significant.

## Results

### LPA increases integrin-FN adhesion

To quantify the cell-bead adhesion, FN-coated beads were applied to the surface of VSMC. The cells, prior to the experiment, were incubated for 2 h in the presence or absence LPA (2 μM). In control cells, not exposed to LPA, the number of adhesion events was 52% lower (*P* < 0.05) than in LPA treated cells (Figure 1E). The analysis of the observed binding events, plotted as histograms of the adhesion forces vs. the corresponding number of adhesion events, revealed that LPA treatment increased the number of adhesion events without affecting the adhesion force (represented by the first peak). The most frequently observed adhesion force was 39 ± 11 pN for control (Figure 1C) and 40 ± 13 pN for LPA (Figure 1D). LPA also induced a rightward shift in the adhesion forces resulting in an increase in the number of adhesion events at higher forces (~80 pN, Figure 1D).

As integrins containing β_1_ and β_3_ subunits are the most common receptors for FN (Johansson et al., 1997), we determined the specificity of binding by incubating VSMC with function-blocking antibodies directed against of β_1_ and β_3_integrins. Incubation of these antibodies with VSMC in the presence of LPA resulted in a significant 65% decrease in the number of adhesions per curve compared with cells exposed to LPA alone (*P* < 0.05, Figure 1F). In contrast, the presence of an iso-type antibody, as a control, in cells exposed to LPA did not reduce the integrin-FN adhesion (Figure 1G). Therefore, the observed adhesion events appear to represent bonds formed between FN and β_1_ and β_3_ containing integrins.

### Ki16425 blocks the LPA-induced increased adhesion of VSMC to FN

**Figure 2:**
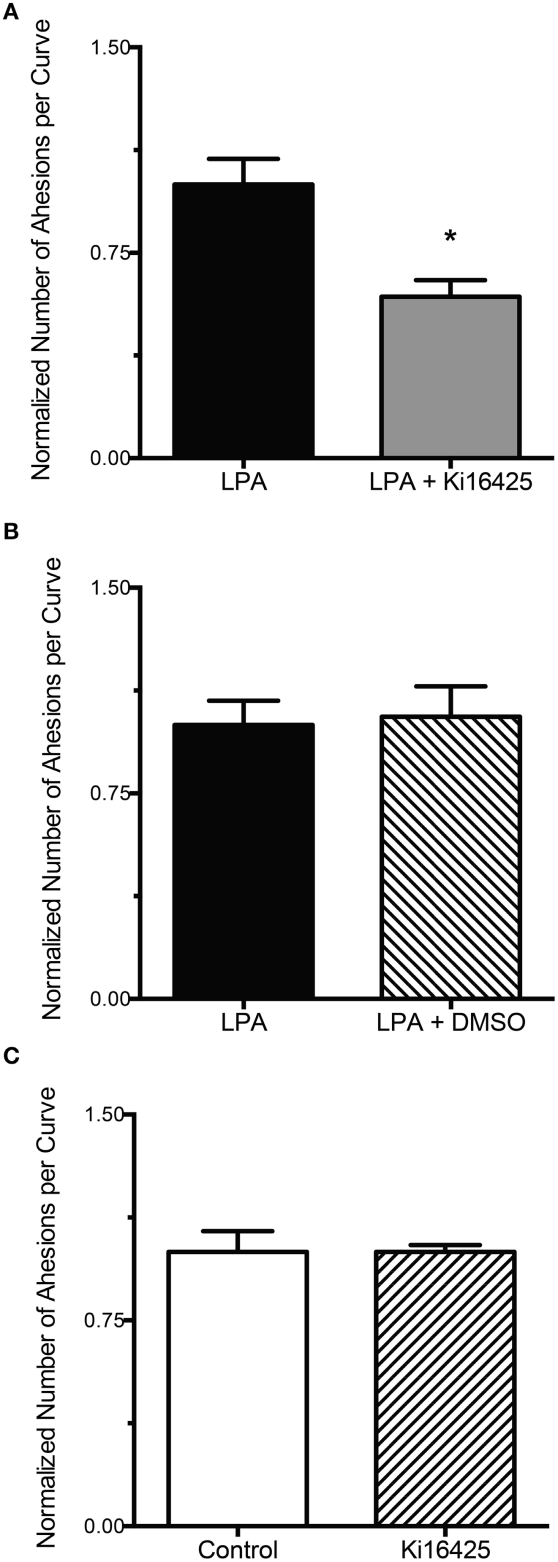
**LPA-induced FN-integrin adhesion is decreased by blockade of LPA receptors LPAR1 and LPAR3**. **(A)** LPA treatment (2 μM) for 2 h in the presence of Ki16425 (10 μM, *n* = 40) results in a 40% decrease in FN-integrin adhesion compared with the cells treated with LPA alone (*n* = 40). **(B)** Incubation of VSMC with LPA (2 μM) in the presence of DMSO (*n* = 39), the solvent for Ki16425, does not result in a change in FN-integrin adhesion compared with VSMC treated with LPA (2 μM) alone (*n* = 39). **(C)** Ki16425 (10 μM, *n* = 40) alone does not change significantly the integrin-FN adhesion of VSMC compared with untreated controls (*n* = 40). Data are mean ± s.e.m. ^*^P < 0.05 vs. LPA alone.

To determine if LPA (2 μM) increased integrin-FN adhesion through its G-protein coupled receptors, experiments were performed in the presence of the LPAR inhibitor Ki16425. Exposure of VSMC to LPA, in the presence of Ki16425 (10 μM), resulted in a significant 40% decrease in the number of adhesions per curve compared with the cells treated with LPA alone (*P* < 0.05, Figure 2A). Incubation of VSMC with DMSO, the vehicle for Ki16425, did not significantly affect the adhesion of VSMC to FN in cells treated with LPA (Figure 2B). Similarly, in VSMC incubated with Ki16425 alone the integrin-FN adhesion was not significantly different from untreated control cells (Figure 2C).

### Tempol or apocynin reduces LPA-induced adhesion of VSMC to FN

**Figure 3:**
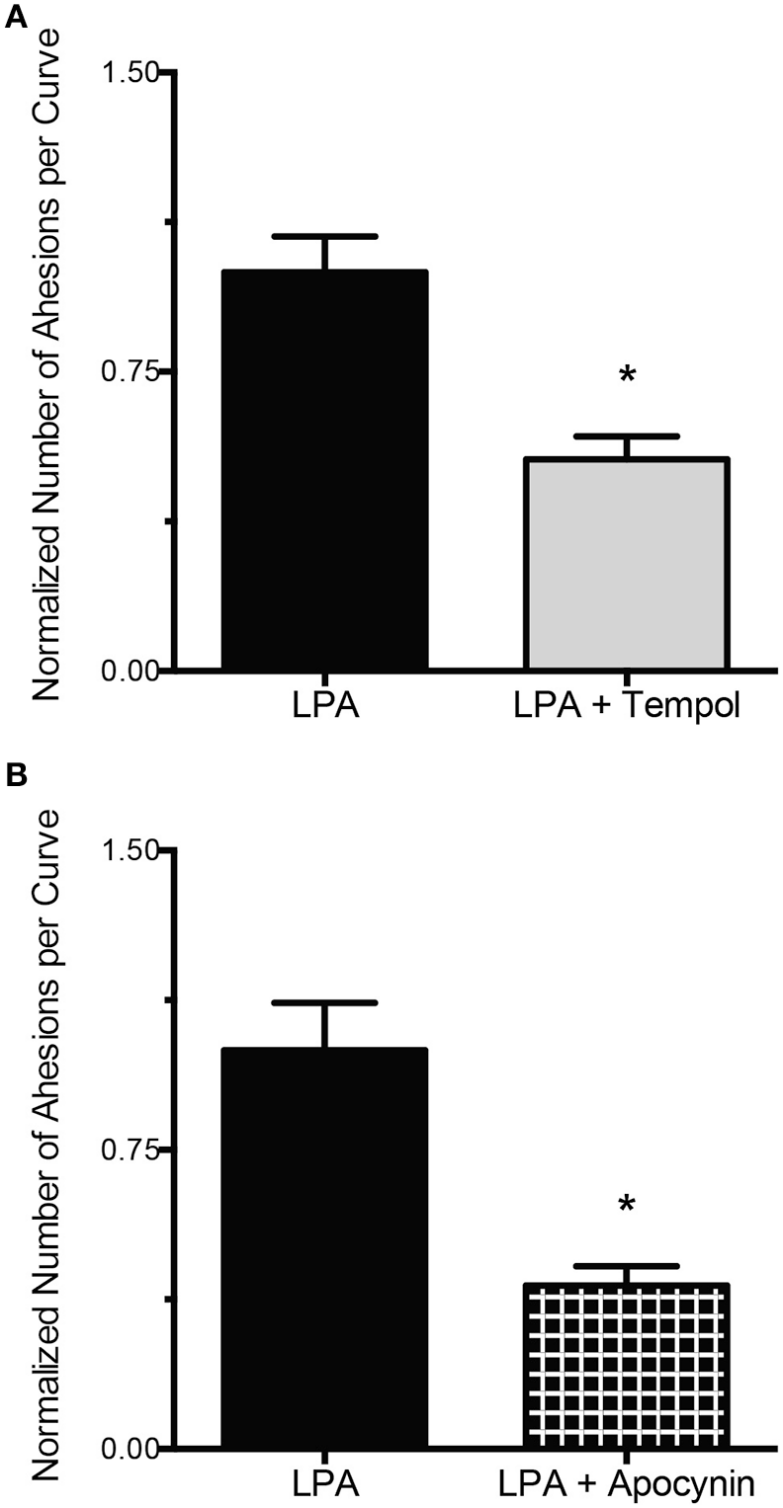
**ROS inhibition abolishes the LPA-induced increase in FN-integrin binding**. **(A)** Incubation of VSMC with LPA (2 μM) for 2 h in the presence of tempol (250 μM, *n* = 36) results in a 47% decrease in FN-integrin adhesion compared with LPA alone (*n* = 35). **(B)** Compared with LPA alone (*n* = 40), 2 h of LPA (2 μM) treatment, in the presence of apocynin (300 μM, *n* = 39), reduced FN-integrin adhesion by 59%. Data are means ± s.e.m. ^*^P < 0.05 vs. LPA alone.

To determine the role of ROS in LPA-induced integrin-FN adhesion, VSMC were incubated with LPA in the presence or absence of the ROS inhibitors tempol or apocynin. In VSMC exposed to LPA and tempol (250 μM) the binding of integrins to FN was decreased by 47% below the level of the VSMC treated with LPA alone (*P* < 0.05, Figure 3A). Furthermore, VSMC exposed to LPA and apocynin (300 μM) also showed a 59% decrease in integrin-FN adhesion compared with VSMC treated with LPA alone (*P* < 0.05, Figure 3B). VSMC incubated 2 h with either tempol or apocynin alone had no significantly changes in integrin-FN adhesion compared to control cells not exposed to LPA (data not shown).

### LPA increases ROS production by VSMC through a mechanism involving LPAR1 and LPAR3

**Figure 4:**
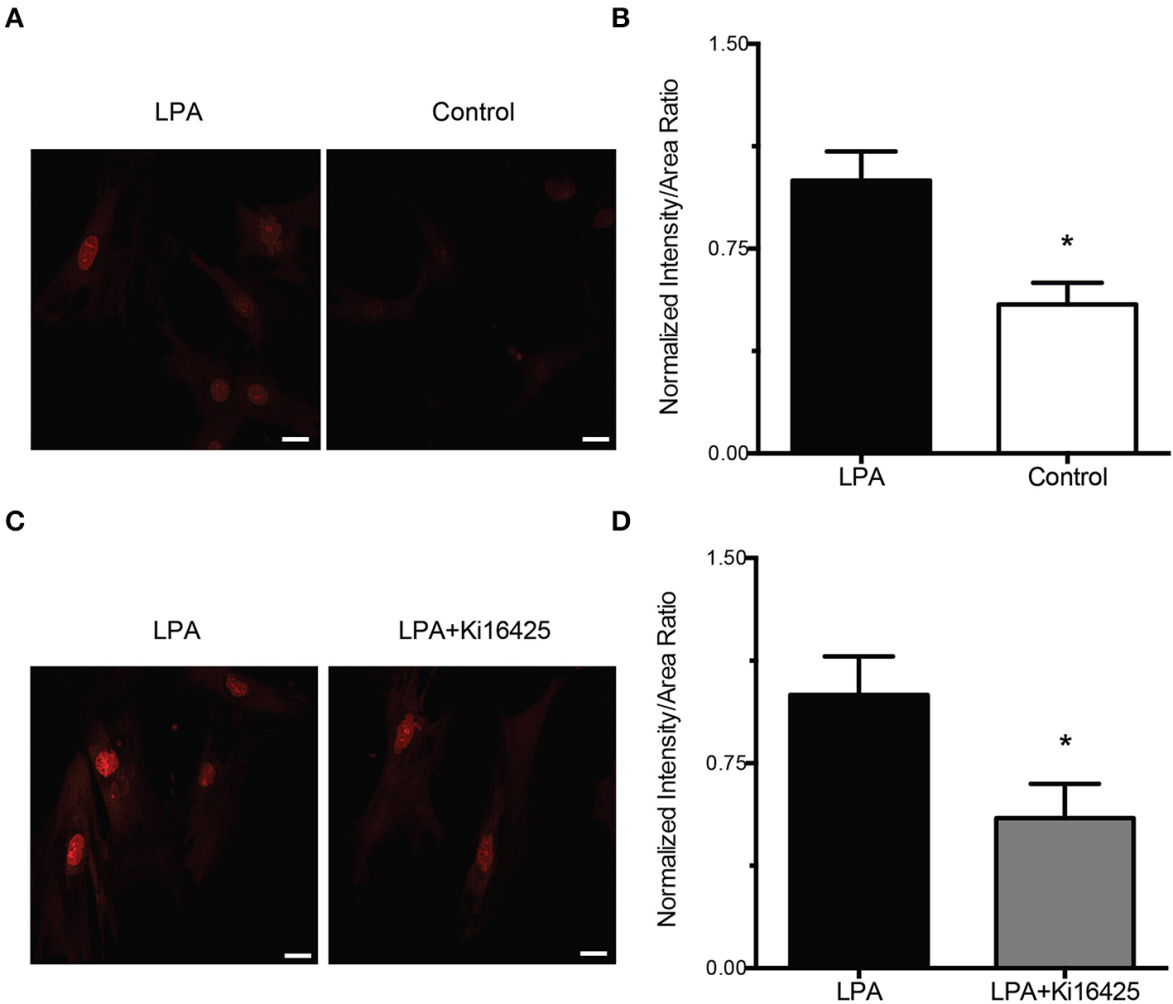
**LPA-induced ROS production is inhibited by blockade of LPAR1 and LPAR3**. **(A)** Representative images of DHE-dependent fluorescence in VSMC exposed to 2 μM LPA (left) or vehicle control (right) for 2 h. **(B)** In VSMC exposed for 2 h to DHE (10 μM, *n* = 5) alone the fluorescence intensity is 45% lower than in VSMC incubated 2 h with LPA + DHE (*n* = 5). **(C)** Representative images of DHE-dependent fluorescence in VSMC exposed LPA alone (left) or LPA + Ki16425 (right). **(D)** VSMC exposure to both Ki16425 (10 μM, *n* = 5) and LPA reduced the increase in fluorescence induced by LPA alone by 45% (*n* = 5). Data were normalized to the average intensity values of LPA treatment. Bar size = 20 μm. Data are means ± s.e.m. ^*^P < 0.05 vs. LPA + DHE.

To confirm that the increase in the production of ROS was induced by LPA, VSMC were incubated in serum free media with LPA and DHE, a superoxide specific fluorescent detector. In control VSMC exposed to DHE (10 μM) alone, after 2 h of treatment, the fluorescent intensity was 45% below the level of the VSMC incubated 2 h with LPA (2 μM) and DHE (10 μM) (*P* < 0.05, Figures 4A,B). Furthermore, to determine that the signaling pathway responsible for the ROS production increase involved the surface receptors LPAR1 and LPAR3, experiments were performed in the presence or absence of Ki16425. In VSMC incubated 2 h with LPA (2 μM) and DHE (10 μM), in the presence of Ki16425 (10 μM), the ROS-associated florescence intensity decreased by 45% (*P* < 0.05, Figures 4C,D), significantly below the fluorescence levels of VSMC exposed for the same amount of time only to LPA (2 μM) and DHE (10 μM), for 2 h (Figure 4D).

### LPA enhances myogenic constriction of isolated resistance arteries

**Figure 5:**
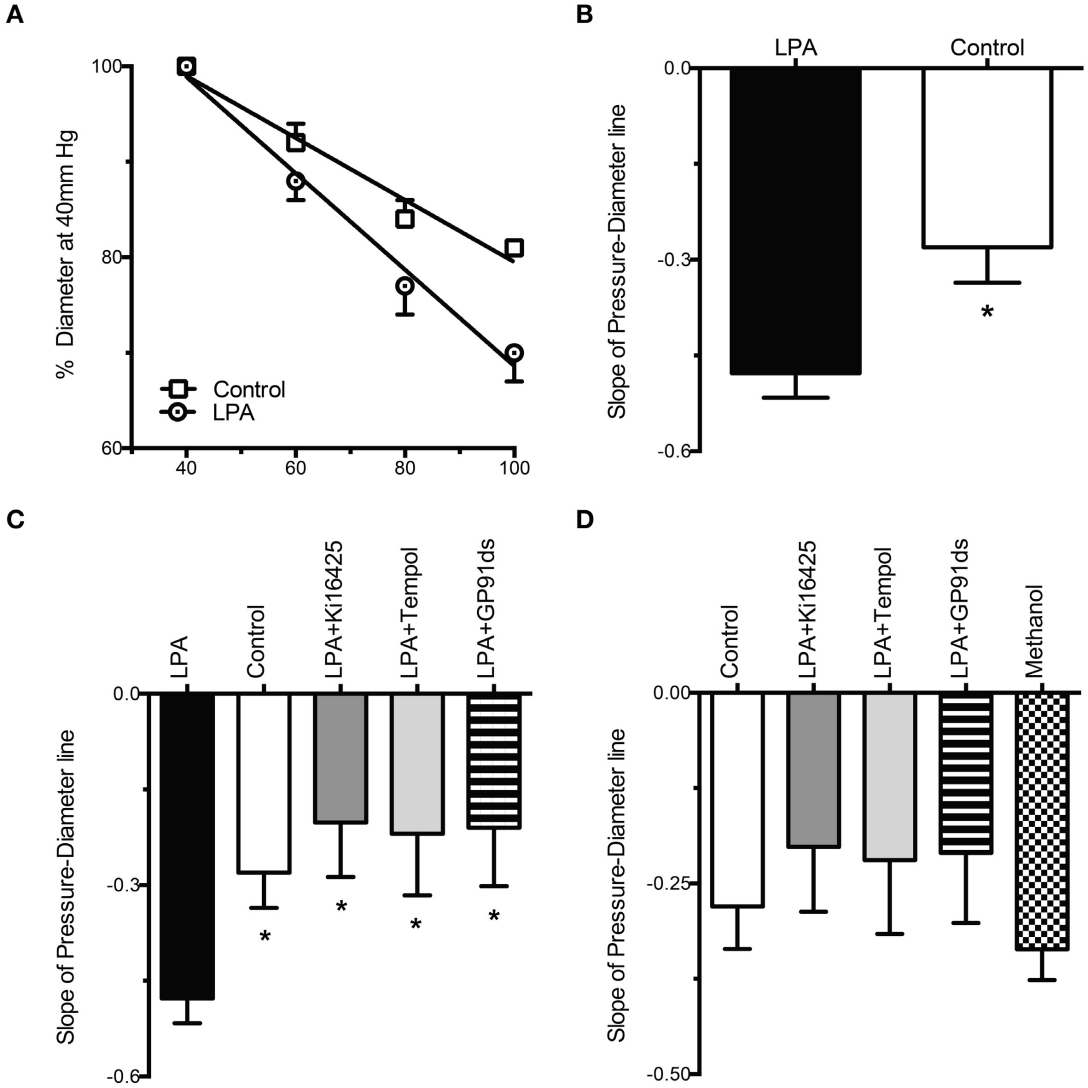
**LPA-induced enhancement of myogenic constriction is blocked by Ki16425, tempol and gp91 ds-tat. (A)** For the same step increases in intraluminal pressure, vessels exposed to LPA (2 μM) for 2 h (*n* = 14) constrict more than control arterioles (*n* = 12). Variations in diameter are expressed as a percent of the vascular diameter attained at 40 mm Hg. **(B)** Slope analysis of the myogenic constriction, determined by the regression analysis of the diameter changes induced by the step increases in intraluminal pressure, reveals that the LPA treated vessels constrict 71% more than controls. **(C)** Slope analysis reveals that Ki16425 (*n* = 9), tempol (*n* = 9), and gp91 ds-tat (*n* = 9) significantly reduce the myogenic constriction enhancement induced by LPA. **(D)** Slope analysis of the myogenic constriction reveals that Ki16425 (*n* = 9), tempol (*n* = 9), and gp91 ds-tat (*n* = 9) reduce the myogenic constriction induced by LPA to control levels. Methanol alone (*n* = 8), the solvent for Ki16425, does not significantly affect the myogenic constriction of resistance arteries. Data are means ± s.e.m. ^*^P < 0.05 vs. LPA **(B,C)**.

Exposure of cremasteric arterioles, for 2 h, to LPA (2 μM) caused a significant enhancement of the myogenic constriction induced by consecutive increments in intraluminal pressure compared with that of control vessels incubated with vehicle alone (Figure 5A). The slope analysis of the pressure vs. diameter regression line revealed that LPA caused a significant 71% increase in myogenic constriction compared with controls (LPA = −0.48 ± 0.04 vs. control = −0.28 ± 0.06, *P* < 0.05, Figure 5B).

### Ki16425 and the blockade of ROS production blunts the LPA-induced enhancement in myogenic constriction

To determine how LPA was enhancing the myogenic constriction, isolated arterioles were incubated for 2 h with LPA (2 μM) in the presence of the LPAR1, LPAR3 inhibitor Ki16425 (10 μM), the superoxide dismutase mimetic tempol (250 μM) and the inhibitor of NADPH oxidase, gp91 ds-tat (50 μM). The slope analysis of the pressure vs. diameter regression line revealed that vessels exposed to LPA in the presence of Ki16425 exhibited a significant 58% decrease in myogenic constriction compared with vessels treated with LPA alone (LPA + Ki16425 = −0.2 ± 0.09 vs. LPA = −0.48 ± 0.04, *P* < 0.05, Figure 5C). Similarly, tempol decreased the myogenic constriction induced by LPA by 56%, significantly different from the vessels exposed to LPA alone (LPA + tempol = −0.22 ± 0.1 vs. LPA = −0.48 ± 0.04, *P* < 0.05, Figure 5C). Vessels exposed to LPA in the presence of gp91 ds-tat also exhibited a significantly reduced myogenic constriction, by 55%, compared with resistance arteries exposed to LPA alone (LPA + gp91 ds-tat = −0.21 ± 0.09 vs. LPA = −0.48 ± 0.04, *P* < 0.05, Figure 5C). There were no significant differences in the slopes of the myogenic constriction between control vessels, not exposed to LPA, and the vessels treated with LPA + Ki16425, LPA + tempol, and LPA + gp91 ds-tat (Figure 5D). Methanol (0.25% v/v), the solvent for Ki16425, did not significantly change the slope of the myogenic constriction compared with control vessel (methanol = −0.34 ± 0.04 vs. control = −0.28 ± 0.06, Figure 5D).

## Discussion

The principal finding of this study is that in rat VSMC the LPA-induced increase in integrin-FN adhesion is dependent on the release of ROS. Based on previous publications indicating that LPA modulates integrin-ECM binding (Sun et al., 2005; Eriksson et al., 2006; Valenick and Schwarzbauer, 2006) and triggers integrin mediated intracellular signaling (Tatler et al., 2011) in cultured cells, we first performed a series of AFM experiments to determine the effects of 2 h of exposure to LPA (2 μM) on the VSMC adhesion to FN. LPA caused a doubling in the number of adhesion events between cells and the FN coated bead. The LPA-induced rise in adhesive events was blocked by the simultaneous incubation with antibodies directed against β_1_ and β_3_ integrins. These results indicate that the LPA-induced adhesion increase is mediated by integrins containing β_1_ and β_3_ subunits. Indeed, integrins containing these β subunits are specific cell surface receptors for FN (Charo et al., 1990; Wu et al., 1993).

LPA is produced by a number of cell types such as fibroblasts, platelets (Pagès et al., 2001), and also by ovarian cancer cells (Pua et al., 2009). In physiological conditions the plasma concentration of LPA varies between 0.1 μM and 1 μM, whereas serum concentrations can sometimes exceed 10 μM (Lin et al., 2010). In pathological conditions the serum LPA concentration can go up to 20 μM (Swarthout and Walling, 2000). Therefore, the concentration of LPA used in this study is within physiological range. LPA appears to function as a pathophysiological modulator in cells of the cardiovascular system by regulating important cellular functions, such as VSMC contraction, stimulation of VSMC proliferation, modulation of endothelial barrier function and platelet aggregation (Smyth et al., 2008). Indeed, exogenous LPA, in rats, elevates arterial blood pressure (Tokumura et al., 1978), while topical application in pigs causes vasoconstriction of cerebral arteries (Tigyi et al., 1995). The finding that LPA induces intimal hyperplasia in conduit arteries (Yoshida et al., 2003) suggests that LPA is involved in VSMC migration and proliferation, processes that are associated with integrin activity and modulation, as well as with ROS production (Gerthoffer, 2007). LPA-stimulated cell migration and proliferation appears to be dependent on the activation of the G-protein coupled receptors LPAR1, LPAR2, and LPAR3 (Kim et al., 2006; Panchatcharam et al., 2008). These findings suggest that LPA signaling through LPAR1-3 might modulate integrin activity, as an increased turnover of focal adhesions is required for enhanced VSMC migration. Thus, our results showing that LPA increases integrin-FN binding in VSMC indicates that LPAR might modulate the process of integrin-FN adhesion.

To determine the role of LPA signaling through its cell surface receptors, we exposed VSMC to LPA in the presence of Ki16425, a specific inhibitor of LPAR1-3 (Ohta et al., 2003). Although, Ki16425 also moderately inhibits LPAR2 receptor (*K_i_* = 6.5 μM), the contribution of this receptor to the LPA-mediated integrin activation, in rat VSMC, is unlikely because of recent studies showing that LPAR2 is not present on the surface of rat VSMC (Subramanian et al., 2010; Zhou et al., 2010). Ki16425 is a short lived reversible antagonist of LPAR1 and LPAR3 (Ma et al., 2009), therefore, to insure continual inhibition of LPAR, the concentration of Ki16425 used in our study was one order of magnitude higher than the *K_i_* = 0.93 μM necessary to inhibit LPAR3 signaling. At the concentration of 10 μM, Ki16425 has been shown to significantly inhibit LPA-induced cell migration (Kim et al., 2008). Our data showed that Ki16425 (10 μM) induced a 40% decrease in integrin-FN adhesion suggesting that LPA-induced increase in integrin-FN adhesion is mediated through a mechanism involving LPAR1 and LPAR3. The decrease in LPA-induced integrin-FN adhesion is comparable to the adhesion levels found in control VSMC, unexposed to LPA. As Ki16425 alone does not change the adhesion of VSMC, our results indicate that the LPA-induced adhesion increase is almost completely blocked by Ki16425.

As mentioned above, LPA-induced SMC growth results in the production ROS, through Rac dependent signaling (Schmitz et al., 2002; Kaneyuki et al., 2007). Also, ROS are important modulators of integrin function (Gregg et al., 2004; Begonja et al., 2005). Cumulatively, the evidence from those studies together with our results, showing that LPA increases integrin binding activity, suggests that LPA might induce increased production of ROS leading to integrin activation. Therefore, to determine whether the increase in integrin-FN adhesion, induced by LPA, is modulated through ROS dependent signaling, we performed experiments in the presence of tempol (250 μM), a superoxide dismutase mimetic, and apocynin (300 μM). We have previously shown that tempol and apocynin at these concentrations are effective scavengers of superoxide (Martinez-Lemus et al., 2011). Our results showed that tempol blunted the LPA-induced integrin-FN adhesion increase, suggesting that production of superoxide anion is associated with the increase in integrin adhesion. Moreover, the incubation of VSMC with LPA in the presence of apocynin also resulted in decreased integrin-FN adhesion. Our results, obtained in VSMC incubated 2 h with LPA in the presence of DHE (a superoxide specific florescent dye) confirmed that LPA (2 μM) increased the production of superoxide. The increase in superoxide production, induced by LPA, was almost completely inhibited by the blockade of LPAR1 and LPAR3 with Ki16425. Overall, these results suggest that, in our experimental conditions, LPA signaling through LPAR1 and LPAR3 induces an enhanced production of superoxide, which modulates, in part, the increase in VSMC integrin adhesion to FN.

Integrins, by linking the ECM and cellular cytoskeleton, have been extensively studied as bi-directional transducers of mechanical and chemical signals across the cell membrane (Hu and Luo, 2013). In this context, integrins have been shown to be important components in the mechanisms responsible for the acute control of vascular diameter by directly modulating the myogenic tone and constriction of isolated resistance arteries (Martinez-Lemus et al., 2005a). In these vessels, myogenic constriction, characterized by vasoconstriction in response to pressure elevation, is believed to significantly contribute to the autoregulation of blood flow in order to sustain physiological tissue perfusion. The myogenic constriction is altered in pathological conditions such as, hypertension and diabetes (Falcone et al., 1993; Hill and Meininger, 1993). Based on our current data and previous results (Martinez-Lemus et al., 2005a), changes in integrin-ECM adhesions should result in an altered myogenic constriction to intraluminal pressure step increases. Therefore, to determine whether LPA altered myogenic constriction, we performed experiments in isolated cremasteric arterioles in the presence of LPA. Our results indicate that LPA enhances myogenic constriction. These results suggest that integrin activation and/or ROS may be responsible, in part, for the observed increase in constriction. This is consistent with previous data showing that integrins are important components in the mechanisms involved in myogenic constriction (Martinez-Lemus et al., 2005a). However, the direct effects of integrin blockade in the presence of LPA cannot be easily tested, as blockade of β_1_ and β_3_ integrins with specific antibodies blunts myogenic constriction (Martinez-Lemus et al., 2005a). Therefore, we performed experiments to determine the effects of LPA, on myogenic constriction, in the presence of Ki16425 (10 μM), tempol (250 μM) and gp91 ds-tat (50 μM). By testing the involvement of LPAR1/LPAR3 and superoxide on myogenic constriction, in vessels exposed to LPA, we would indirectly evoke our findings from VSMC, where blockade of LPA signaling and ROS scavenging reduced integrin activation. We expected that by blocking the mechanisms of LPA-induced integrin activation we would observe a reduction in the LPA-enhanced myogenic constriction. Our results confirm that indeed, the blockade of LPA signaling through LPAR1 and LPAR3, with Ki16425, reduced the slope of myogenic constriction to control levels. Additionally, superoxide scavenging with tempol also resulted in a reduction in myogenic constriction, similar to control levels. Further inhibition of ROS release from NADPH oxidase with apocynin was not performed, as the role of apocynin as a specific inhibitor of NADPH oxidase has been challenged. A couple of studies suggest that in VSMC apocynin predominantly acts as an antioxidant to reduce ROS (Heumuller et al., 2008; Schlüter et al., 2008). Therefore, to test the specific involvement of NADPH oxidase and the production of superoxide in the LPA-induced increase in myogenic constriction, we used the NADPH oxidase inhibitor gp91 ds-tat. We found that blockade of NADPH oxidase activity with gp91 ds-tat reduced the LPA-induced increase in myogenic constriction to control levels as tempol did. These data are consistent with previous work showing that ROS production increases myogenic constriction (Ren et al., 2010). Our results, from resistance vessels, indicate that LPA is inducing an increased myogenic constriction though mechanisms that include LPAR1 and LPAR3, as well as the generation of superoxide anion. These results corroborate those obtained in isolated VSMC and suggest that LPA, through its LPAR1 and LPAR3, increases NADPH oxidase-dependent superoxide production. Although it could be argued that the effects of tempol acting as a SOD mimetic should increase production of H_2_O_2_, tempol is known to possess catalase-like actions that rapidly convert H_2_O_2_ into water (Wilcox and Pearlman, 2008). In this study we did not measure the amount of H_2_O_2_ produced by cells or vessels in the presence of tempol, but our results using the specific NADPH oxidase inhibitor gp91 ds-tat strongly suggest that it is indeed superoxide the main ROS responsible for modulating the myogenic response. The release of ROS, in turn, results in the activation of integrins and promotes integrin-ECM adhesions. Therefore, LPA, by increasing integrin-FN adhesion, appears to modulate myogenic constriction in resistance vessels.

Our findings clearly identify ROS as modulators of VSMC integrin activity and the myogenic response. This knowledge increases our understanding of the process of mechanotransduction that occurs at the level of VSMC in resistance arteries and their response to changes in intraluminal pressure. However, a number of questions on the identity of the mechanosensors responsible for detecting changes in intraluminal pressure and the sequence of events that ultimately change the level of VSMC contraction and vascular diameter remain to be fully elucidated. We have previously shown that blockade of α_v_β_3_ and α_5_β_1_ integrins inhibits myogenic vasoconstriction in resistance vessels and have speculated on their potential role as mechanosensors (Martinez-Lemus et al., 2003, 2005a,b). A recent study, however, clearly indicates that the angiotensin-II type 1_a_ (AT-1) receptor is mechanosensitive and that its absence dampens the myogenic response in resistance arteries (Schleifenbaum et al., 2014). As stimulation of the AT-1 receptor is known to induce ROS production, it could be speculated that stretch sensitive G-protein coupled receptors may be the mechanosensors responsible for inducing the production of ROS that subsequently activates integrins in the myogenic response. In this scenario, the role of ROS-dependent integrin activation could be associated with: (1) an increase in cell adhesion as reported in the present study, (2) the strengthening of actin cytoskeletal structures as it has been reported occurs in VSMC following angiotensin-II stimulation (Hong et al., 2014), and (3) actin polymerization as it has been shown occurs during myogenic vasoconstriction (Cipolla et al., 2002; Flavahan et al., 2005). In addition, the specific sources of ROS also remain to be clearly identified. Our present results suggest that the main source is NADPH oxidase. However, a recent study suggests that mitochondrial superoxide and H_2_O_2_ may also be involved in the myogenic process (Gebremedhin et al., 2013). If that is the case, the mechanisms by which mitochondria are induced to produce ROS in response to increments in vascular intraluminal pressure would need to be elucidated. Our current findings also relate to previous reports indicating that integrins and ROS are involved in the phenotypic changes experienced by VSMC in different pathological circumstances such as, hypertension and atherosclerosis. In the microcirculation, we have recently shown that the inward remodeling of resistance arteries, commonly encountered in essential hypertension is associated with the repositioning of VSMC and the production of ROS (Martinez-Lemus et al., 2004, 2011). We also found that inhibition of the small GTPase Rac1, a component of the active NADPH oxidase, blocks the development of this specific type of remodeling (Staiculescu et al., 2013). Additionally, in the small arteries of (mRen2) 27 rats, inward eutrophic remodeling is dependent on the activity of α_V_β_3_ integrins (Heerkens et al., 2006). Moreover, the vascular remodeling triggered by infusion of LPA is associated with VSMC proliferation and migration (Cheng et al., 2009), and integrins modulate some of these adaptive changes. Indeed, α_5_β_1_ integrins control in part the migration and proliferation of VSMC within the atherosclerotic plaque (Barillari et al., 2001). Cumulatively, those studies and our results are consistent with current evidence indicating that ROS and LPA modulate cellular signaling pathways that induce both acute and chronic changes in VSMC behavior via integrins. Our experiments, in isolated arterioles, also suggest that LPA might play a role in the modulation of vasomotor behavior through a mechanism involving the release of ROS and integrin activation. A better understanding of the integrin modulation by LPA through ROS dependent pathways should provide new therapeutic approaches for controlling the adverse cardiovascular events associated with atherosclerosis and hypertension.

## Author contributions

Luis A. Martinez-Lemus, Gerald A. Meininger, and Marius C. Staiculescu conceived and designed the study, and analyzed and interpreted the data. Marius C. Staiculescu, Francisco I. Ramirez-Perez, and Jorge A. Castorena-Gonzalez performed experiments, analyzed the data, and made figures. Zhongkui Hong and Zhe Sun performed experiments, analyzed data and interpreted data. Marius C. Staiculescu wrote the manuscript. Luis A. Martinez-Lemus, Gerald A. Meininger, Francisco I. Ramirez-Perez, Jorge A. Castorena-Gonzalez, Zhongkui Hong, and Zhe Sun edited the manuscript. All authors approved the final version of the manuscript.

### Conflict of interest statement

The authors declare that the research was conducted in the absence of any commercial or financial relationships that could be construed as a potential conflict of interest.

## Abbreviations

VSMC: vascular smooth muscle cell
ECM: extracellular matrix
ROS: reactive oxygen species
LPA: lysophosphatidic acid
LPAR: lysophosphatidic acid receptor
AFM: atomic force microscopy
TGF-β: transforming growth factor-β
NADPH: nicotinamide adenine dinucleotide phosphate
PSS: physiological saline solution
DHE: dihydroethidium.
